# Versatility of the *Burkholderia cepacia* Complex for the Biosynthesis of Exopolysaccharides: A Comparative Structural Investigation

**DOI:** 10.1371/journal.pone.0094372

**Published:** 2014-04-10

**Authors:** Bruno Cuzzi, Yury Herasimenka, Alba Silipo, Rosa Lanzetta, Gianfranco Liut, Roberto Rizzo, Paola Cescutti

**Affiliations:** 1 Department of Life Sciences, University of Trieste, Trieste, Italy; 2 Department of Chemical Sciences, University of Naples Federico II, Naples, Italy; University of Malaya, Malaysia

## Abstract

The *Burkholderia cepacia* Complex assembles at least eighteen closely related species that are ubiquitous in nature. Some isolates show beneficial potential for biocontrol, bioremediation and plant growth promotion. On the contrary, other strains are pathogens for plants and immunocompromised individuals, like cystic fibrosis patients. In these subjects, they can cause respiratory tract infections sometimes characterised by fatal outcome. Most of the *Burkholderia cepacia* Complex species are mucoid when grown on a mannitol rich medium and they also form biofilms, two related characteristics, since polysaccharides are important component of biofilm matrices. Moreover, polysaccharides contribute to bacterial survival in a hostile environment by inhibiting both neutrophils chemotaxis and antimicrobial peptides activity, and by scavenging reactive oxygen species. The ability of these microorganisms to produce exopolysaccharides with different structures is testified by numerous articles in the literature. However, little is known about the type of polysaccharides produced in biofilms and their relationship with those obtained in non-biofilm conditions. The aim of this study was to define the type of exopolysaccharides produced by nine species of the *Burkholderia cepacia* Complex. Two isolates were then selected to compare the polysaccharides produced on agar plates with those formed in biofilms developed on cellulose membranes. The investigation was conducted using NMR spectroscopy, high performance size exclusion chromatography, and gas chromatography coupled to mass spectrometry. The results showed that the Complex is capable of producing a variety of exopolysaccharides, most often in mixture, and that the most common exopolysaccharide is always cepacian. In addition, two novel polysaccharide structures were determined: one composed of mannose and rhamnose and another containing galactose and glucuronic acid. Comparison of exopolysaccharides obtained from cultures on agar plates with those extracted from biofilms on cellulose membranes showed important differences, thus suggesting that extrapolating data from non-biofilm conditions might not always be applicable.

## Introduction

Bacterial exopolysaccharides are commonly biosynthesized by microbial species and can be grouped in two main types: capsular polysaccharides and extracellular polysaccharides (**EPOLs**) [1]. The formers are strongly bound to bacterial cell forming a capsule, while the latter are excreted in the medium around bacteria, often forming a slime. Several biological functions are attributed to **EPOLs**, but not all are fully known. Certainly, they contribute to create a suitable hydrophilic niche where bacteria may more comfortably survive, by providing accumulation of micronutrients. Furthermore, they have protective functions by forming a physical barrier around cells and exhibiting more or less specific interactions with environmental molecules potentially dangerous for the bacteria. In this context, also environmental conditions in infection sites have to be considered, where bacteria are threatened by components of the immune system, such as antimicrobial peptides [2] and reactive oxygen species [3], [4]. It can thus be reasonable to assume that bacteria use **EPOLs** as versatile tools for their survival. As a matter of fact, a single species is often able to synthesize a variety of **EPOLs** with distinct primary structures, and therefore diverse conformations, thus implying potential different biological properties.

Besides the above considerations, an interesting bacterial way of life, the biofilm, is deeply connected to **EPOL** functions. **EPOLs** are considered one of the main component of bacterial biofilms [5]–[7], but it has to be taken into account that biofilm composition is rather variable and depends on several parameters including culture media and the nature of the support to which cell adhesion occurs. In fact, in biofilms **EPOLs** are often considered as part of the tools used by bacteria for cell adhesion. Moreover, anti-biofilm and anti-adhesive properties of some **EPOLs** have recently been described [8], thus increasing the versatility of these macromolecules in terms of biological properties. It has to be stressed that, as pointed by Høiby et al. [9], only biofilms produced by *Pseudomonas aeruginosa* and *Staphylococcus aureus* have been deeply investigated, but a more general definition of matrix properties based on a wide number of bacterial species is still lacking. For the above reasons, microbiological investigations aimed at the description of the interactions of bacteria with their surroundings, in the environment as well as in infections, should imply an accurate knowledge of the structure of extracellular polysaccharides, as one of the main molecular characteristic of bacteria.

In the frame of an investigation on bacteria involved in lung infections of cystic fibrosis (CF) patients, and with the aim of developing a study devoted to disclose the **EPOLs** role in biofilm formation and maintenance, we thoroughly investigated the structure and the macromolecular properties of **EPOLs** produced by some members of the *Burkholderia cepacia* Complex (*BCC*) [1]. *BCC* includes several species of the genus *Burkholderia* which are currently found in many environmental niches where they also have beneficial effects for plant protection and growth [10], [11]. Contrary to this, some species of the genus are pathogens for plant, animals and vulnerable humans, specifically CF and Chronic Granulomatous Disease patients. The ability of some *BCC* species to cause serious lung infections in CF patients was first reported in 1984 and since then the number of *BCC* species found in CF lungs increased substantially. Contrary to other CF pathogens, some *BCC* infections lead to a rapid fatal decline known as “cepacia syndrome”. Until now the *BCC* was described to include eighteen species out of more than eighty characteristic of the genus, but the taxonomical definition of the *BCC* is still an open task [12]–[14]. We then decided to investigate 9 species (Table 1) which include environmental bacteria as well as those more frequently involved in lung infection of CF patients. Most of them are part of the suggested reference panel [12], [13] defined by members of the “International *Burkholderia cepacia* Working Group” (http://users.ugent.be/~tcoenye/index_bestanden/index_files/Page383.htm), while some others are type strains. Although these species are often the subject of studies implying properties related with **EPOLs** functions, there is a rather poor information on the structure of **EPOLs** produced by these 9 species; very often it is taken for granted that they produce the already described Cepacian (**CEP**) [1] as the main **EPOL**.

**Table 1:** *BCC* species investigated for the structure of their **EPOLs**.

Species	LMG*	Other strain designation	
*B. ambifaria*	19466	AU0212	Not ref. panel, not type strain
*B. anthina*	20983	C1765	ref panel [13]
*B. cenocepacia*	16659	C1394	ref. panel [12]
*B. cenocepacia*	18829	PC184	ref. panel [12]
*B. cepacia*	18821	CEP 509	ref. panel [12]
*B. dolosa*	21820	R12720	type strain
*B. lata*	22485	b383	type strain
*B. multivorans*	16660	C1576	ref. panel[12]
*B. stabilis*	18138		Not ref. panel, not type strain
*B. vietnamiensis*	10929		ref. panel [12]

*BCCM/LMG  =  Belgian Co-ordinated Collections of Micro-organisms/Laboratory of Microbiology, Gent University, Belgium.

The investigation described in this paper was carried out resorting to NMR spectroscopy, gas-chromatography coupled to mass spectrometry (GC-MS) and high performance size exclusion chromatography (HP-SEC). It clarified the interesting versatility of this group of bacteria in terms of extracellular polysaccharides biosynthesis. In addition, preliminary data on matrix composition indicated that biofilm growth conditions can modulate **EPOLs** biosynthesis leading to production of a selected polymer or to quantitative changes of **EPOLs** molar ratio in case of mixtures, with respect to the non-biofilm mode of growth.

## Materials and Methods

### Bacterial strains and exopolysaccharides production and purification

Strains to be investigated were acquired from the Belgian Co-ordinated Collections of Micro-organisms at the Laboratory of Microbiology, Gent University, Belgium (Table 1). The strains *B. cenocepacia* BTS2, *B. pyrrocinia* BTS7 and *B*. *cepacia* BTS13, isolated from cystic fibrosis patients attending the Regional Centre for Cystic Fibrosis in Trieste, Italy [15], were used for the production of known **EPOLs**. Bacterial growth on Yeast Extract-Mannitol (YEM) agar plates, as a continuous layer, and **EPOLs** isolation and purification were performed as previously described [16]. Five independent **EPOL** extractions were performed with BTS2, twenty with BTS7 and six with BTS13 (these two strains are used as cepacian and galactan-Kdo producer, respectively), and two with all other strains.

### Biofilm quantification in microtiter plates

*B. cenocepacia* BTS2 and *B. multivorans* C1576 were grown overnight in liquid Müller Hinton (MH) medium at 30°C with shaking and subsequently diluted to a concentration of 1×10^6^ CFU/mL. A volume of 200 μL was used to inoculate the wells in a 96 wells microtiter plate. After incubating at 30°C for 48 h, bacterial growth was evaluated by reading the turbidity at 590 nm, while biofilm biomass was estimated at 570 nm after crystal violet staining [17]. The biofilm index, which is the ratio between the biomass and the cells growth, after subtraction of the proper blank, was used to quantify the biofilm.

### Biofilm production on cellulose membranes

Biofilms were grown on cellulose membranes (Sigma, cut-off 12.400 Da) [17] which were prepared as follows: they were cut in circles to match the Petri dish, washed first in boiling 5% Na_2_CO_3_ and then in boiling water for 15 min, autoclaved and placed over Petri dishes, containing YEM or MH medium. Membranes covered the whole plate and excess of water was let to dry under the hood. An overnight liquid culture of bacteria (*B. cenocepacia* BTS2 or *B. multivorans* C1576) was diluted to 0.013 OD at 600 nm (about 1×10^6^ CFU/mL) and 10 μL of the diluted suspension were placed on the membranes. The liquid medium used for the overnight culture was the same of the seeded Petri dish. After 7 days of incubation at 30°C, the material on the membranes was recovered in 5–10 mL of 0.9% NaCl, centrifuged at 4000 rpm at 4°C for 20 min, and finally filtered sterilised (Millipore membranes 0.22 μm). When YEM medium was used, separation of the cells was achieved by centrifuging at 20000 rpm at 4°C for 30 min, due to the high viscosity of the **EPOL** solution. Removal of proteins was achieved by treatment with protease (from *Streptomyces griseus*, Sigma) in TRIS-HCl 50 mM, pH 7.5. The EPOLs were purified by dialysis, first against 0.1 M NaCl and then water, the solutions were then taken to neutral pH and filtered. The extractions were repeated three times for both species.

### General procedures for structural analysis

Hydrolysis of the polysaccharides was conducted with 2 M TFA for 1 h at 125°C and alditol acetates were prepared as already described [18]. Permethylation of the **EPOL** was achieved following the protocol by Harris [19], while carboxyl reduction was performed with NaBD_4_ as previously described [20]. Analytical gas-chromatography (GC) was performed on a Perkin–Elmer Autosystem XL gas chromatograph equipped with a flame ionisation detector and an SP2330 capillary column (Supelco, 30 m), using He as carrier gas. The following temperature programmes were used: for alditol acetates, 200–245°C at 4°C/min; for partially methylated alditol acetates, 150–250°C at 4°C/min. GC–MS analyses were carried out on an Agilent Technologies 7890A gas chromatograph coupled to an Agilent Technologies 5975C VL MSD. In the case of overlapping peaks of partially methylated alditol acetates, GC-MS was performed also on a HP-1 column (Agilent Technologies, 30 m) using the temperature program 120–245°C at 2°C/min.

### NMR spectroscopy

The molecular mass of **EPOLs** was decreased by treating their solutions (1 g/L) with a Branson sonifier equipped with a microtip at 2.8 Å. Samples were cooled in an ice bath and sonicated using 5 bursts of 1 min each, separated by 1 min intervals. They were subsequently exchanged three times with 99.9% D_2_O by lyophilisation and finally dissolved in 0.7 mL 99.96% D_2_O. Spectra were recorded on a 500 MHz VARIAN spectrometer operating at 50°C. Chemical shifts are expressed in ppm using acetone as external reference (2.223 ppm for ^1^H). NMR spectra were analysed using Mestrenova software.

### Molecular mass determination

High performance size exclusion chromatography (HP-SEC) was performed on an Agilent Technologies 1200 series HPLC equipped with three columns in series (Tosoh Bioscience, TSKgel G3000PW, G5000PW and G6000PW, i.d. 7.5 mm, length 30 cm) kept at 40°C with a thermostat (Waters Millipore). Calibration of the chromatographic system was performed using pullulan standards (Polymer Laboratories, Germany and Sigma for pullulan with MM = 1.6×10^6^). Elution was performed with 0.15 M NaCl, with a flow rate of 0.4 mL/min and monitored using a refractive index detector (Knauer, Labservice Analytica), interfaced with a computer via Agilent software.

## Results and Discussion

The knowledge already acquired on the structure of the **EPOLs** produced by some *BCC* species, and in particular on the structural fingerprints given by 1D ^1^H-NMR spectra, prompted the use of this technique to define the **EPOLs** produced by the selected species for the present investigation. Besides NMR, classical monosaccharide composition and glycosidic linkage analyses were also used to gain structural information on novel **EPOLs**. In addition, since the biological activity depends also on the dimension of the macromolecules, **EPOLs** molecular masses were conveniently investigated by means of HP-SEC. The data acquired were compared with those already available in our laboratory and in the literature.

In order to facilitate the following discussion on polysaccharides structure, the NMR data of the **EPOLs** produced by three *BCC* clinical isolates investigated in our laboratory, *B. cenocepacia* BTS2, *B. pyrrocinia* BTS7, and *B. cepacia* BTS13 [21] are hereafter summarised, since they were particularly useful for the present study. Moreover, NMR data for BTS2 and BTS7 polysaccharides were never published before.

### EPOLs produced by *B. pyrrocinia* BTS7

**Figure 1:**
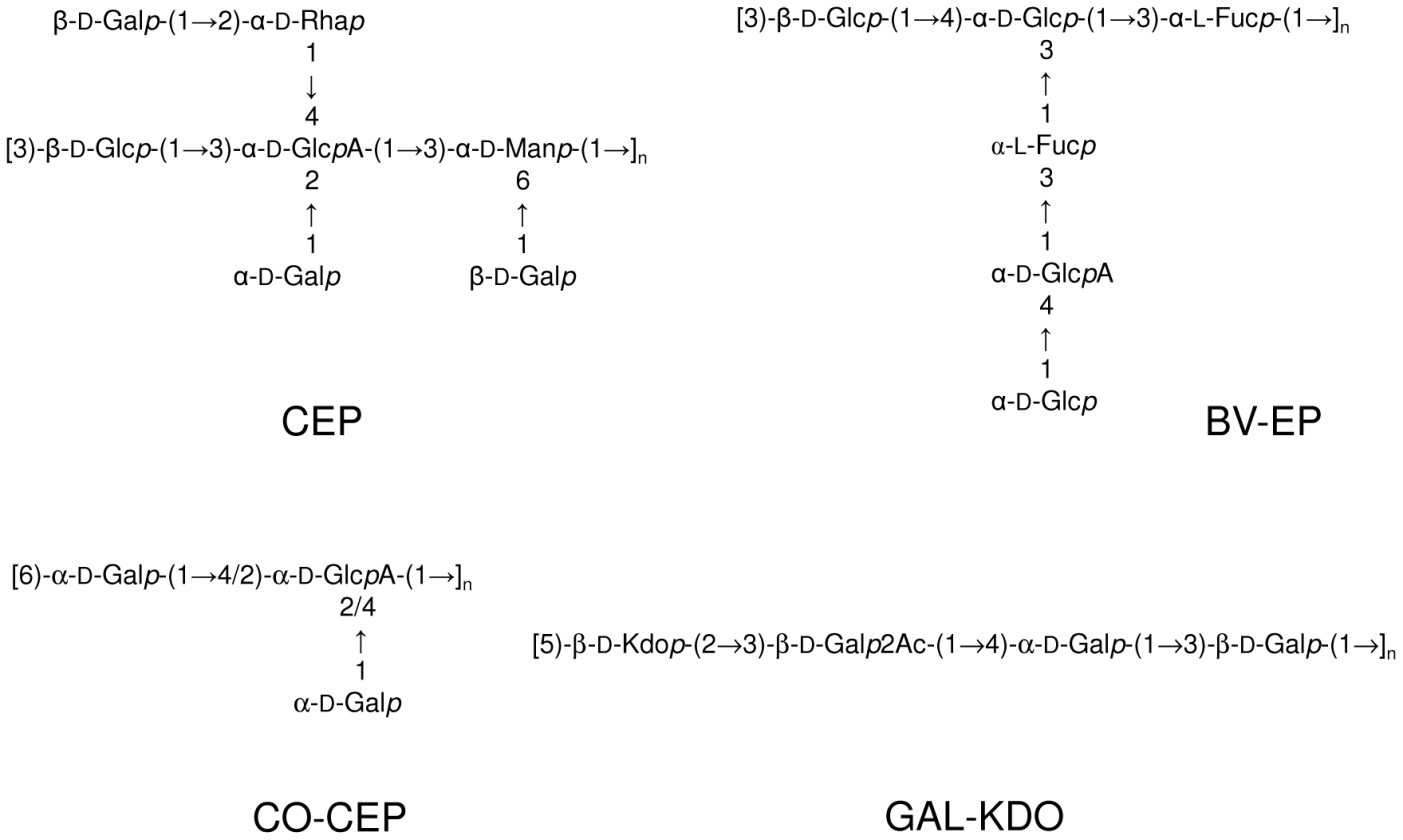
Repeating units structure of exopolysaccharides produced by *BCC* species. Primary structures of the repeating units of the exopolysaccharides **CEP**, **GAL-KDO**, **BV-EP** and **CO-CEP**.

**Figure 2:**
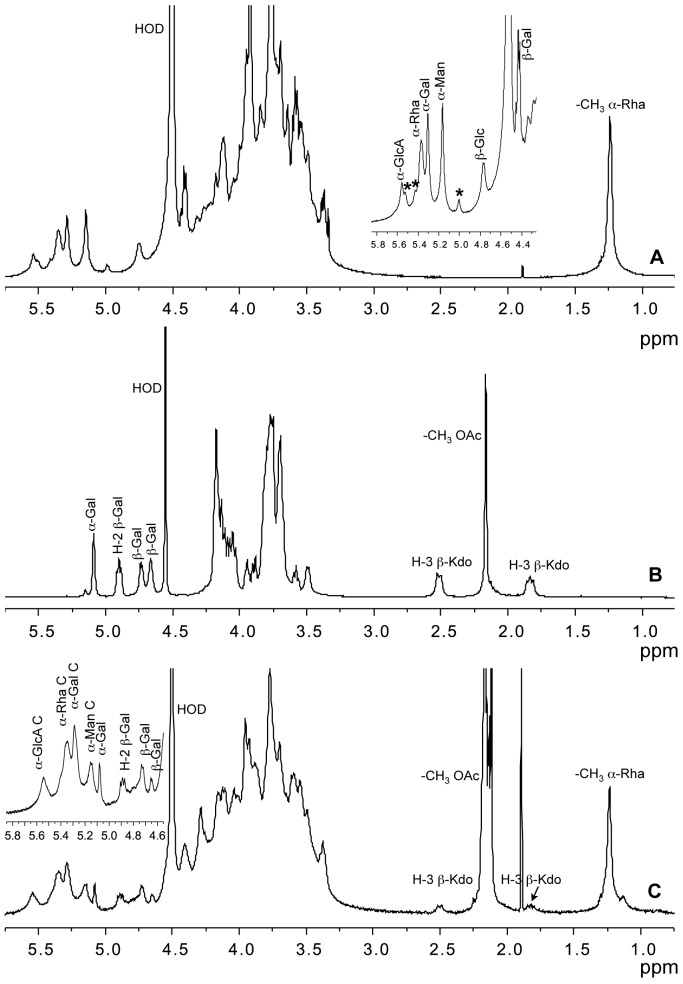
^1^H-NMR spectra of exopolysaccharides produced by *BCC* species. A) ^1^H-NMR spectrum of de-acetylated **CEP** produced by *B. pyrrocinia* BTS7. The anomeric proton region is enlarged in the inset. Anomeric proton resonances of **CEP** are indicated, asterisks refer to signals of **CO-CEP**. B) ^1^H NMR spectrum of **GAL-KDO** produced by *B. cepacia* BTS13 grown on YEM agar plates: anomeric proton resonances together with H-2 of acetylated β-Gal and H-3 of Kdo are indicated. C) ^1^H NMR spectrum of the **CEP**/**GAL-KDO** mixture produced by *B. cenocepacia* BTS2 grown on YEM agar plates. The anomeric proton region is enlarged in the inset; signals belonging to **CEP** are indicated with the suffix C. Methyl groups of O-acetyl esters (-CH_3_ OAc) and rhamnose residues (-CH_3_ α-Rha) are shown.

*B. pyrrocinia* BTS7 produces the **EPOL** named cepacian (**CEP**), which is constituted by a complex repeating unit and contains glucose, mannose, galactose, the unusual D-rhamnose together with a glucuronic acid fully substituted on its hydroxyl groups (Figure 1). Prior to NMR, the sample was de-acetylated and sonicated, to enhance the resolution of the spectrum. In the ^1^H NMR spectrum (Figure 2A), anomeric proton assignments are reported in agreement with previous articles [22]–[24]. In the same ppm range, the small signals indicated with asterisks at 5.51, 5.41 and 4.99 ppm could not be attributed neither to **CEP**, nor to any other known **EPOL** of the *BCC*, thus suggesting the presence of a novel **EPOL** hereafter referred to as **CO-CEP**. Different polysaccharide productions always contained both **EPOLs**, with **CO-CEP** in very small amounts (less than 0.10 **CO-CEP**/**CEP** repeating units molar ratio). Separation of the two **EPOLs** by gel filtration chromatography was attempted (data not shown), but it was unsuccessful, probably due to the viscous nature of **CEP** solution and to its aggregation ability. The small amounts of **CO-CEP** produced by strain BTS7 always in mixture with **CEP** did not permit the determination of its structure.

### EPOLs produced by *B. cepacia* BTS13

*B. cepacia* BTS13 produced two different **EPOLs**, depending on the medium used [21]: a linear polysaccharide constituted of three galactose residues, one acetyl substituent and one 3-deoxy-D-manno-oct-2-ulosonic acid (Kdo), named galactan-Kdo (**GAL-KDO**), (Figure 1, GAL-KDO) when grown on King A, and a mixture of the former with a linear 2,6-linked β-fructan, known as levan, in YEM medium. The ^1^H-NMR spectrum of **GAL-KDO** is shown in Figure 2B where anomeric proton resonances together with signals due to acetylation are indicated. Moreover, the two H-3 resonances of the Kdo residue are found at about 2.50 and 1.80 ppm, well separated from the other signals, thus being diagnostics for the presence of such residue.

### EPOLs produced by *B. cenocepacia* BTS2

This clinical isolate produced a mixture of **GAL-KDO** and **CEP** when grown on YEM agar plates, as established by NMR spectroscopy analysis (Figure 2C). Identification of the NMR resonances was achieved by comparison with the NMR spectra of the individual polysaccharides described in the previous paragraphs. The anomeric region of the ^1^H-NMR spectrum (Figure 2c) was rather crowded, but the resonances of **CEP** were clearly visible. On the contrary, the anomeric protons of **GAL-KDO** were not well resolved, since they partially overlapped with H-1 of 3-linked Glc of **CEP** and with other resonances due to acetylation. However, the presence of **GAL-KDO** was undoubtedly established by the H-3′s signals at 2.51 and 1.83 ppm. Integration of the peak areas belonging to H-3′s of Kdo and that one of C-6 of Rha (1.24 ppm) of **CEP** repeating unit established a ratio of about 0.18 for the two **EPOLs**.

### Composition analysis of the EPOLs produced by the investigated strains

The overall composition homogeneity of the **EPOLs** produced by the 10 investigated isolates, belonging to the 9 selected species, was determined by GC analysis of their alditol acetates derivatives and the results were compared with data available in the literature for **CEP** (Table 2) [23], the most common exopolysaccharide of the *BCC*. Out of the ten products examined, only two **EPOLs** had a substantially different composition: the one from *B. vietnamiensis*, containing fucose residues, and the one from *B. multivorans* C1576, characterised by a relative molar ratios of saccharides quite different from **CEP**. The composition of all other polysaccharides did not show striking differences and indicated mainly the production of **CEP**.

**Table 2:** Determination of neutral monosaccharides in the **EPOLs** of different *BCC* bacterial isolates and comparison with literature data for **CEP** [23].

	monosaccharide^a^				
Species	Rha	Fuc	Man	Gal	Glc
*B. ambifaria*	0.99		0.16	2.69	1.00
*B. anthina*	0.92		0.64	2.43	1.00
*B. cenocepacia* 16659	0.74		0.52	2.48	1.00
*B. cenocepacia* 18829	0.77		0.79	3.06	1.00
*B. cepacia*	0.91		0.48	3.12	1.00
*B. dolosa*	0.80		0.34	2.05	1.00
*B. lata*	0.92		0.42	2.44	1.00
*B. multivorans*	2.22		1.64	2.60	1.00
*B. stabilis*	0.62		0.34	2.07	1.00
*B. vietnamiensis*	0.31	0.23	0.08	1.00	1.00
Cepacian [23]	0.71		0.50	2.79	1.00

a Monosaccharide content is expressed as molar ratio relative to glucose.

### ^1^H-NMR spectroscopy and linkage determination of the EPOLs produced by the investigated strains

The **EPOLs** produced by all strains were investigated by 1D ^1^H-NMR spectroscopy in their native as well as de-acetylated forms. The spectra showed that they all produced **CEP**, confirming that its biosynthetic gene cluster is widely distributed within the *BCC*. NMR spectroscopy revealed also that many isolates were able to biosynthesise polysaccharides different from **CEP**. In fact, the production of **CEP** was almost invariably partnered by **CO-CEP**, except in the case of *B. multivorans* C1576. *B. anthina, B. ambifaria*, and the two strains of *B. cenocepacia* C1394 and 18829 exhibited the highest **CO-CEP** co-synthesizing ability. The ratio between **CO-CEP** and **CEP** in these species was in the range 0.1-0.3, as measured by integrating the anomeric proton signals at 4.76 ppm, belonging to 3-linked Glc in **CEP**, and the one at 4.98 ppm, attributed to **CO-CEP**. Due to the impossibility of isolating this polysaccharide, its structural investigation was carried out on the **EPOLs** mixture produced by *B. ambifaria*, characterised by a rather high amount of **CO-CEP**. 2D-NMR spectroscopy investigation (data not shown) was not decisive, because of signals overlapping. Useful information was obtained with methylation analysis, performed on the native as well as the carboxyl reduced **EPOLs** mixture. The results (Table 3) revealed the presence of two novel residues, besides the expected ones composing **CEP**: 1,6-linked-Gal and 1,2,4-linked-Hex. Due to reduction with NaBD_4_, the latter derivative was di-deuterated on C-6, thus identifying it with an hexuronic acid in the native polysaccharide. The lack of 1,2,4-linked standard hexoses to determine the identity of the branched hexuronic acid was filled by comparing the retention time of several branched hexoses available in the lab with published data [25]. The results suggested to identify it with glucose establishing that native **CO-CEP** contains 1,2,4-linked GlcA. Moreover, the lack of a terminal non-reducing residue different from t-Gal, suggested that t-Gal itself could be the substitution on the branched glucuronic acid. These three residues represent the composition of **CO-CEP** and are in agreement with the presence of three NMR signals at 5.51, 5.41 and 4.99 ppm (signals with asterisks in Figure 2). A tentative structure is then constituted of a disaccharidic backbone 6)-α-D-Gal*p*-(1→4/2)-α-D-GlcA*p*-(1→ with a terminal α-D-Gal*p* substituting carbon 2 or 4 of the glucuronic acid residue. The position of the side chain substitution could not be assigned.

**Table 3:** Determination of glycosidic linkages in the **EPOLs** of *B. ambifaria* before (Ba) and after carboxyl reduction (Ba-R), and *B. multivorans* C1576 separated on SP2330 (Bm-I) and on HP1 (Bm-II) columns.

Linked residue^a^	RRT^b^	Ba	Ba-R	Bm-I	Bm-II	RRT^c^
2-Rha	0.91	0.72	0.64	1.00	1.00	0.84
3-Rha	0.93			0.43	0.43	0.86
t-Gal	1.00	2.27	2.11	1.71	1.82	1.00
3-Glc	1.13	1.00	1.00	0.68		
2-Man+3-Man	1.14			0.92		
3-Glc+2-Man					1.31	1.17
3-Man					0.47	1.20
3-Gal	1.17	0.20	0.21	0.13	0.17	1.22
6-Gal	1.30	0.20	0.19			
2,4-GlcD_2_ d	1.40		0.09			
3,6-Man	1.43	0.39	0.50	0.30	0.29	1.51
2,3,4-GlcD_2_ d	1.47	-	0.21	-	-	

a Position of glycosidic linkages.

b Relative retention time on SP2330 column.

c Relative retention time on HP1 column.

d C6 di-deuterated according to GC–MS.

*B. cepacia*, *B. ambifaria*, *B. dolosa*, and *B. stabilis* were able to co-synthesize also the **GAL-KDO** exopolysaccharide, as revealed by the presence of the diagnostic resonances of Kdo H-3′s at about 2.50 and 1.80 ppm (data not shown). *B. cepacia* showed the highest **GAL-KDO/CEP** repeating units molar ratio (0.3), followed by *B. stabilis* (0.03), and by the other isolates which had a content below 0.03.

**Figure 3:**
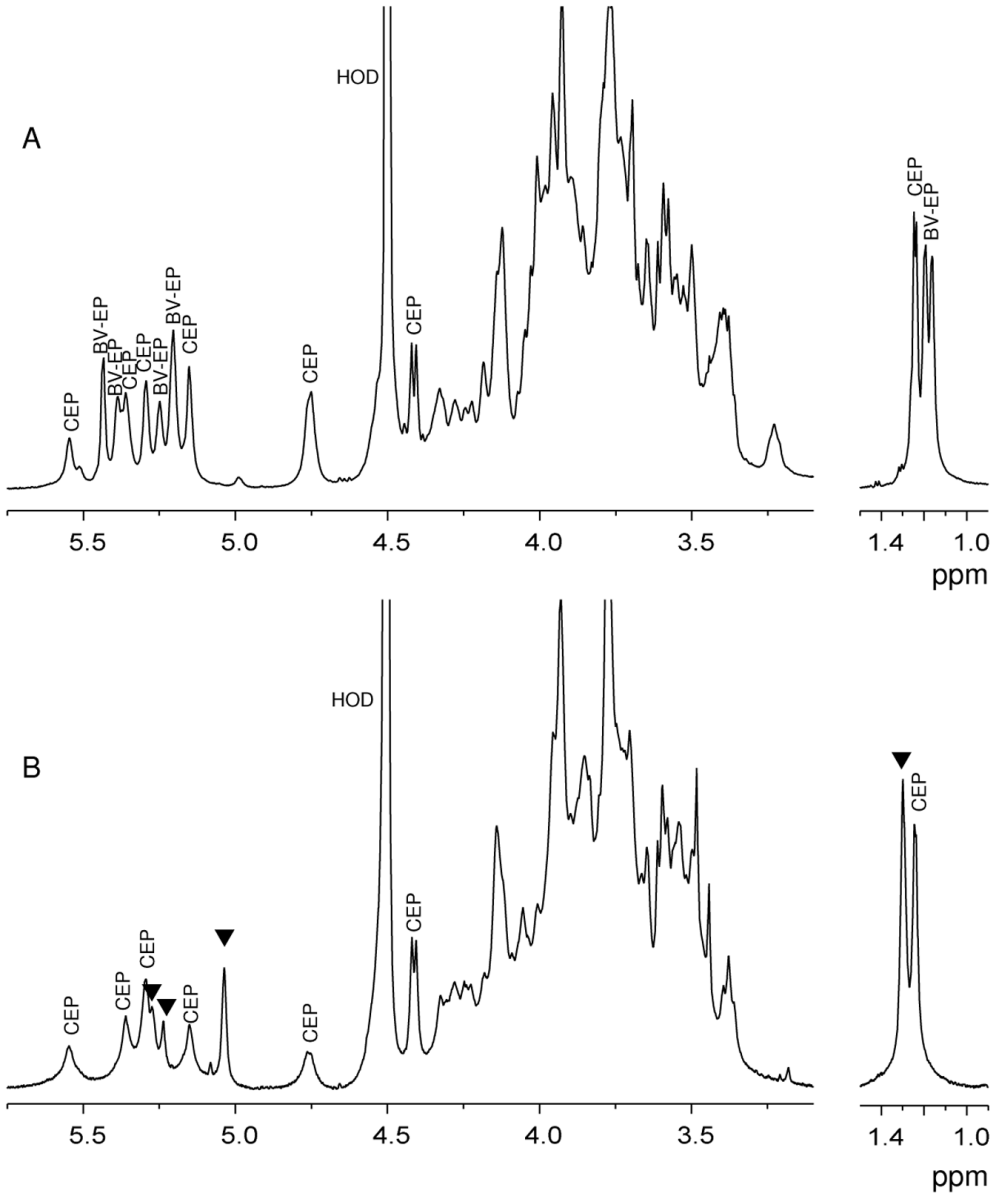
^1^H-NMR spectra of the exopolysaccharides produced by *B. vietnamiensis* and *B. multivorans*. A) ^1^H-NMR spectrum of the **EPOLs** mixture produced by *B. vietnamiensis* on YEM agar plates. Anomeric proton signals and methyl groups of 6-deoxy sugars belonging to **CEP** and **BV-EP** are shown. B) ^1^H-NMR spectrum of the **EPOLs** mixture produced by *B. multivorans* C1576 on YEM agar plates: anomeric proton signals and methyl groups of 6-deoxy sugar of **CEP** are indicated, while triangles indicate new signals.

A remarkable case of co-synthesis is *B. vietnamiensis* which produced two distinct **EPOLs**: **CEP** and the one named **BV-EP** (Figure 1) whose structural definition was reported in a separate article [26]. The ^1^H-NMR spectrum of the mixture **CEP** and **BV-EP** is reported in Figure 3A. **BV-EP** is totally different from **CEP** and it is the only **EPOL** of the *BCC* to contain fucose residues, a feature generally not very common in bacterial polysaccharides. Out of three independent growths and extractions, **CEP** and **BV-EP** were co-produced twice in the same molar ratio, as revealed by ^1^H-NMR spectra (data not shown), while a third batch contained only the polysaccharide **BV-EP**. This is the only case observed in our studies of such a drastic change in **EPOL** biosynthesis; usually the types of polysaccharides produced are constant and, in the case of mixtures, the ratio of the components may vary only slightly.

Considering *B. multivorans* C1576, the **EPOLs** proton anomeric region (Figure 3B) showed resonances belonging to **CEP** together with new signals at 5.27, 5.24 and 5.04 ppm, indicated with triangles. In addition, two distinct –CH_3_ resonances attributed to H-6′s of rhamnose were found at 1.30 and 1.24 ppm, indicating the presence of at least another Rha, besides that one in **CEP**. The ratio of the area of the two peaks was 1.2, indicating roughly equimolar amounts of rhamnose in the two polymers. These findings were in agreement with the composition analysis (Table 2), where rhamnose and mannose residues were more abundant than in **CEP** alone [23], thus suggesting the biosynthesis of a novel **EPOL**. In order to gain structural information, the mixture of *B. multivorans* C1576 **EPOLs** was subjected to methylation analysis and the results are reported in Table 3. Besides the typical residues attributable to **CEP**, 3-linked rhamnose, 2-linked mannose and 3-linked mannose were also present. In addition, the 2-linked rhamnose was present in higher amount than found in **CEP**, indicating that it could also be part of the novel **EPOL**. Quantitative data were impeded by co-elution of 2-linked Man and 3-linked Man when GC was performed on the SP-2330 column, as revealed by the e.i. mass spectrum. However, using the HP-1 column, 3-linked Man was well separated, while co-elution resulted for 2-linked Man and 3-linked Glc (Table 3). Since the integration for all well separated derivatives on both columns was very similar, the amount of 2-linked Man was obtained with a simple subtraction to give: 2-Man = 0.49 and 3-Man = 0.43, relative to 2-Rha. These data are in good agreement with the NMR results and indicate that *B. multivorans* C1576 produces, besides **CEP**, a novel rhamnomannan (**RHA-MAN**) constituted of 2-linked Rha, 3-linked Rha, 2-linked Man and 3-linked Man in equimolar amounts. More structural information on this **EPOL** was obtained during the preliminary investigation of the carbohydrate fractions in biofilms, as described in the last paragraph of the "Results and Discussion" section.

### Macromolecular characterization

**Figure 4:**
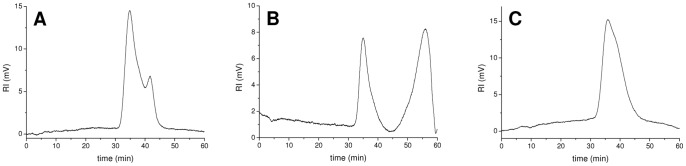
HP-SEC chromatograms of exopolysaccharides mixtures. **EPOL** mixtures produced by *B. multivorans* C1576 (A), *B. vietnamiensis* (B) and *B. cenocepacia* 18829 (C).

Besides primary structure, the overall conformation of a polymer depends on its dimensions. The **EPOL** molecular mass has a strong impact on the biological properties, particularly those related to the structure of the matrix in biofilms, a cellular scaffold filled with water and other useful macro- and micro-molecules. Therefore, the weight average molecular masses of the **EPOLs** produced by *BCC* strains were determined by HP-SEC analysis and the values are reported in Table 4 together with the polydispersity index (D) values. This index is the ratio between the weight- and the number-average molecular mass and indicates the degree of molecular masses dispersion for a given polymeric species: the higher the figure the higher the dispersion. It is interesting to note that only the **EPOLs** mixture produced by *B. multivorans* C1576 and *B. vietnamiensis* exhibited a bimodal elution profile, as illustrated in Figure 4. For the **EPOLs** of the former species, peak assignment was based on the relative amounts of the two polysaccharide, with **RHA-MAN** being less abundant than **CEP** (about 0.5 repeating units molar ratio), as indicated by integration of the C-6′s of rhamnose residues in the ^1^H-NMR spectrum. Therefore, the most intense peak, characterised by a higher MM was attributed to **CEP**, while the less intense one, having a lower MM, was assigned to **RHA-MAN** (Figure 4A). In the case of *B. vietnamiensis* (Figure 4B), **CEP** was characterised by a lower molecular mass than **BV-EP,** as established by composition analysis of the two well separated fractions [26]. Contrary to this, the presence of the **CO-CEP** polymer was not accompanied by extra peaks in the HP-SEC experiments, the only exception was the HP-SEC trace obtained for the **EPOLs** of *B. cenocepacia* 18829, where a shoulder on the right side of the main polymer elution (Figure 4c) might be attributed to **CO-CEP**, based on the relative abundance of the two **EPOLs**. The co-production of **GAL-KDO** did not result in a bimodal distribution or in the appearance of a shoulder in the HP-SEC chromatograms.

**Table 4:** Weight average molecular masses (MM, Da) and polydispersity index (D) of the **EPOL** fractions produced by the investigated *BCC* species.

	Peak I		Peak II	
Species	MM (Da)	D	MM (Da)	D
*B. ambifaria* LMG19466	2.56×10^5^	1.84		
*B. anthina* LMG20983	2.34×10^6^	3.42		
*B. cenocepacia* LMG16659	8.49×10^6^	4.38		
*B. cenocepacia* LMG18829	8.99×10^6^	6.68		
*B. cepacia* LMG18821	1.80×10^7^	4.94		
*B. dolosa* LMG21820	9.63×10^6^	5.79		
*B. lata* LMG22485	1.19×10^5^	2.40		
*B. multivorans* LMG16660	2.09×10^7^	2.48	4.98×10^5^	1.05
*B. stabilis* LMG18138	7.57×10^6^	4.18		
*B. vietnamiensis* LMG10929	1.43×10^7^	3.61	2.06×10^4^	1.93

Concerning numerical values of the molecular masses, all the polymeric fractions investigated had rather high figures. These data might reflect the aggregation capacity exhibited by **CEP** which forms complexes with low stoichiometry (two- or four-stranded complexes) as already reported in previous articles [27], [28]. Therefore, the real values of molecular masses for single **CEP** polymeric chains might be lower than those detected.

### EPOLs produced in biofilms

In a preliminary study of biofilm matrix composition, the structure of **EPOLs** produced by two species of the *BCC* was investigated. The focus was centred on *B. multivorans* C1576 and *B. cenocepacia* BTS2, both producing mixtures of **EPOLs** on YEM agar plates. The latter strain was selected because it is an abundant biofilm producer, as indicated by crystal violet staining when cultured in microtiter plates (data not shown).

**Figure 5:**
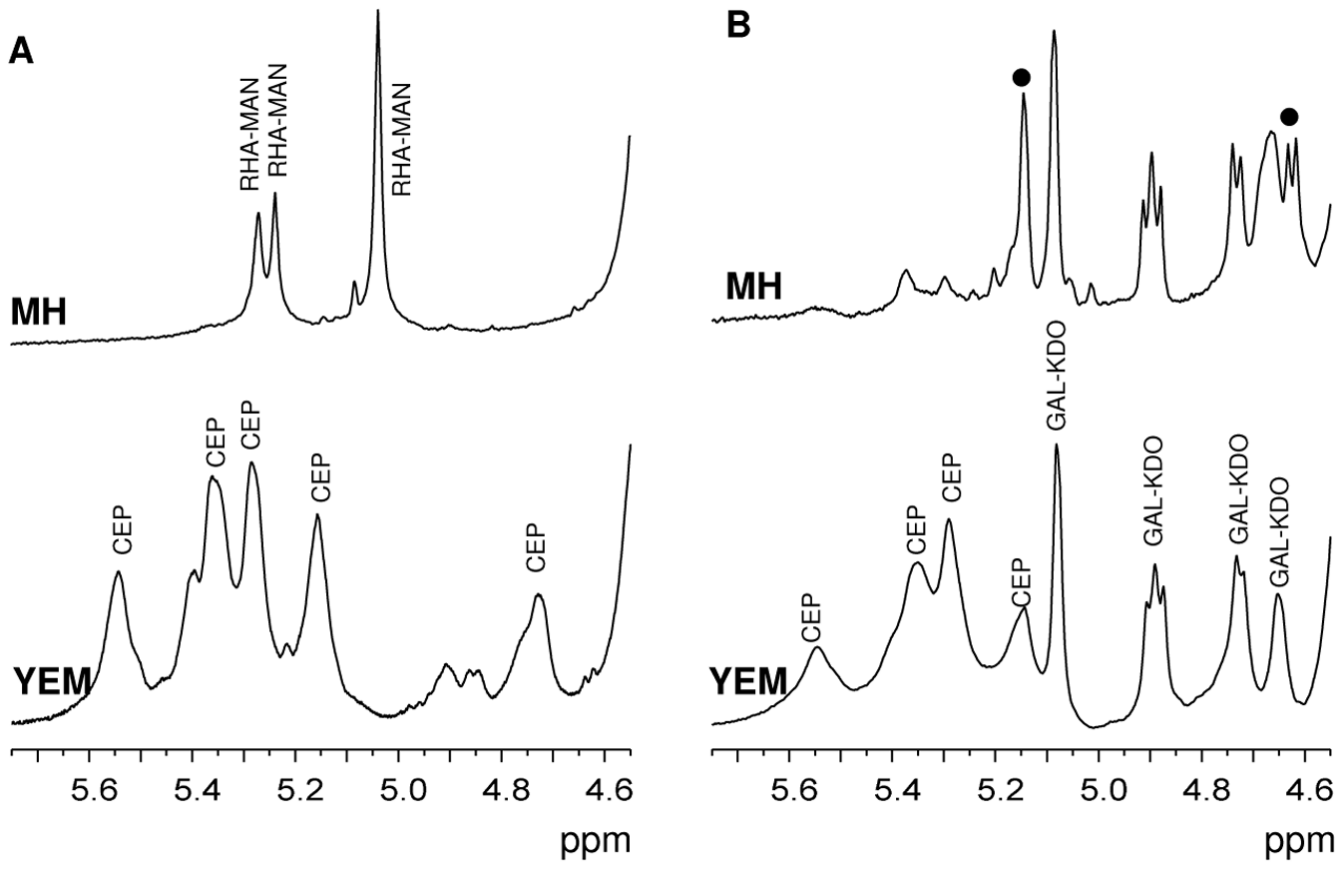
^1^H-NMR spectra of exopolysaccharides from biofilms. A) anomeric region of the ^1^H-NMR spectra of the **EPOLs** obtained from biofilms of *B. multivorans* C1576 produced on cellulose membranes deposited on MH (top) and YEM (bottom) agar plates; B) anomeric region of the ^1^H NMR spectra of **EPOLs** produced by *B. cenocepacia* BTS2 in biofilm conditions in MH (top) and YEM (bottom) media. **CEP**, **GAL-KDO** and **RHA-MAN** indicate signals belonging to the respective polysaccharides. Circles indicate new resonances of unknown identity.

Biofilms were grown for seven days on cellulose porous membranes placed on top of agar plates, containing the appropriate culture medium, either YEM or MH. As previously described, in non-biofilm conditions (i.e. classical agar plates), on YEM medium C1576 produced a mixture of **CEP** and a novel **EPOL** named **RHA-MAN** (Figure 3, Table 3). Surprisingly, ^1^H-NMR spectra showed that in biofilm-forming conditions, YEM medium stimulated only the biosynthesis of **CEP**, whilst MH medium induced only production of the **RHA-MAN** as shown in the proton NMR spectrum (Figure 5A). In fact, the anomeric region showed three resonances at 5.27, 5.24 and 5.04 ppm, having integration values of 1.0: 1.0: 2.0. At 1.30 ppm a signal attributable to a methyl group of 6-deoxyhexose gave an integration value of 6∶0, thus suggesting the presence of two rhamnose residues. Composition analysis indicated that it contained rhamnose and mannose in equimolar amounts, thus undoubtedly identifying it with the **RHA-MAN** **EPOL,** co-produced with **CEP** when bacteria were grown on YEM medium agar plates in non-biofilm conditions.

Therefore, the biofilm culture conditions drove C1576 to produce specific polysaccharides as a function of the medium employed. This observation is very interesting since it brings to the conclusion that **EPOLs** produced in biofilms developed on cellulose membranes might be very different from those biosynthesized during growth on solid agar medium. This conclusion was also achieved by investigating the clinical isolate *B. cenocepacia* BTS2. This isolate produced a mixture of **CEP** and **GAL-KDO** (Figure 2C) when grown directly on agar. However, in biofilm-forming conditions, the mixture was produced only in YEM medium where an increase in the amount of **GAL-KDO** was observed (0.60 **GAL-KDO**/**CEP** repeating units), while MH stimulated the production of **GAL-KDO** with only scanty amount of **CEP** (Figure 5B). At the same time, two new resonances with considerable intensities were detected in the ^1^H NMR spectrum at 5.14 and 4.62 ppm (indicated with circles), thus showing the production of a further novel **EPOL**.

## Conclusions

More than two decades ago, Sage and co-workers [29] described the slime produced by CF isolates of *Burkholderia cepacia*, at that time still named *Pseudomonas cepacia*, as being composed of galactose, glucose, mannose, glucuronic acid, and rhamnose, when grown on excess of glucose or mannitol as carbon source, thus suggesting the presence of **CEP**. Since then, the research in this field made great progress: the *BCC* was established as a group composed of at least eighteen closely related species [12]–[14] and detailed structural studies on the biosynthesised **EPOLs** appeared in the literature. **CEP** resulted to be the main exopolysaccharide of the *BCC*, especially when bacteria were grown on YEM medium and always in non-biofilm conditions [16], [30]. Besides **CEP**, other **EPOLs** were described: **PS-I** was first found in France [31] and afterwards in our laboratory [30]; dextran was produced together with **CEP** and **PS-I** by a *B. cenocepacia* clinical isolate investigated in Canada [31], and levan, a fructose polymer, was detected in our laboratory, co-synthetized with **GAL-KDO** by *B. cepacia* BTS13 [21]. Very recently, *B. vietnamiensis* **EPOL** was determined to have a hexasaccharidic repeating unit containing fucose, glucose and glucuronic acid [26]. The results reported in this paper further contributed to unravel the complex picture of the polysaccharide biosynthetic potential of the *BCC*, but not all the details were clarified so that additional studies should be carried out. In the present study, **CEP** was confirmed to be the **EPOL** produced on YEM medium by the majority of the species of the *BCC* in non-biofilm conditions. Moreover, eight species investigated co-synthesized another extracellular polysaccharide (**CO-CEP**) containing t-Gal, 6-Gal and 1,2,4-GlcA. *B. multivorans* C1576 co-synthesized a novel linear **EPOL** containing 2-Rha, 3-Rha, 2-Man and 3-Man in equimolar amounts (Table 5).

**Table 5:** Summary of the different **EPOLs** produced by each bacterial species when grown on YEM solid medium.

Species	LMG	CEP	GAL-KDO	CO-CEP	BV-EP	RHA-MAN
*B. ambifaria*	19466	**+**	**+**	**+**		
*B. anthina*	20983	**+**		**+**		
*B. cenocepacia*	16659	**+**		**+**		
*B. cenocepacia*	18829	**+**		**+**		
*B. cepacia*	18821	**+**	**+**	**+**		
*B. dolosa*	21820	**+**	**+**	**+**		
*B. lata*	22485	**+**		**+**		
*B. multivorans*	16660	**+**				**+**
*B. stabilis*	18138	**+**	**+**	**+**		
*B. vietnamiensis*	10929	**+**			**+**	

Some chemical features are common to the majority of the **EPOLs** investigated. Specifically, the molecular mass is usually larger than 10^6^ Daltons and the polymer chains often bear negative charges due to the presence of carboxylate groups, mostly uronic acids, thus foreseeing a certain degree of chain rigidity due to charge repulsion.

Considering the species investigated in biofilm conditions, it must be underlined that the biosynthesis of **CEP** was stimulated by YEM medium both in non-biofilm and in biofilm mode of growth. However, when biofilm was formed on cellulose membranes deposited on MH agar plates, polysaccharides other than **CEP** were prevalent. This is an important information underlying that the type of **EPOLs** synthesised not only varies with the medium used, but it is also influenced by the presence of the solid support.

In conclusion, the species of the BCC are characterised by an extremely high variability in the **EPOLs** production. Taking into account that the presence of the extracellular polysaccharidic matrix might be strongly influenced by the bacteria environmental conditions, the **EPOL** identity should be carefully considered for microbiological investigations including biofilm formation ability, where **EPOL** might have a specific role in controlling the hydrophilic matrix surrounding bacterial cells and modulating its properties in response to external conditions.
